# A curated and integrated dataset for exploring global bee-plant interactions

**DOI:** 10.1038/s41597-026-06970-5

**Published:** 2026-03-07

**Authors:** Sajad Noori, Alice C. Hughes, Thais N. C. Vasconcelos, John S. Ascher, Jared T. Miller, Sarah M. Gaugel, Madeleine M. Ostwald, James B. Dorey, Victor H. Gonzalez, Aline C. Martins, Michael C. Orr, Katja C. Seltmann

**Affiliations:** 1 State Museum of Natural History Stuttgart, Stuttgart, Germany; 2 KomBioTa-Center for Biodiversity and Integrative Taxonomy, University of Hohenheim and State Museum of Natural History, Stuttgart, Germany; 3 Department of Biosciences, University of Melbourne, Melbourne, Australia; 4 Department of Ecology and Evolutionary Biology, University of Michigan, Michigan, USA; 5 Department of Biological Sciences, National University of Singapore, Singapore, Singapore; 6 Florida Museum of Natural History, Department of Biology, University of Florida, Gainesville, Florida USA; 7 School of Biological and Behavioural Sciences, Queen Mary University of London, London, UK; 8 Environmental Futures Research Centre, School of Science, University of Wollongong, Wollongong, New South Wales Australia; 9 Biodiversity Institute and Natural History Museum, Department of Ecology & Evolutionary Biology, University of Kansas, Lawrence, Kansas USA; 10 Instituto Nacional de Pesquisas da Amazônia (INPA), Manaus, Brazil; 11 Cheadle Center for Biodiversity and Ecological Restoration, University of California, Santa Barbara, California USA

**Keywords:** Entomology, Biodiversity

## Abstract

Bees are one of the most important pollinators in terrestrial ecosystems, supporting biodiversity and food production. However, global knowledge of their interactions with host plants remains limited. To address this, we describe and refine a subset of the Global Biotic Interactions (GloBI) database focused on bee-plant interactions. We updated taxonomy using current checklists and enhanced the dataset with metadata on geography, endemism, and human uses of plants. The resulting dataset includes 981,982 unique interaction records between 5,537 bee species and 12,699 plant taxa. Despite its scale, the dataset is affected by strong taxonomic and geographic biases. It covers only 26% of described bee species and 4% of flowering plant taxa—primarily those used by humans—and is heavily skewed toward North America and Western Europe. Nevertheless, GloBI represents a valuable resource for incorporating bee-plant interactions into biodiversity and conservation-oriented research and represents a considerable advance in our current knowledge.

## Background & Summary

Large-scale biodiversity studies require integrated, interoperable, and standardized data to assess and mitigate the ongoing biodiversity crisis^1,2^. Establishing platforms to pool and harmonize existing and unpublished data is a first step in determining and filling gaps in our knowledge of biodiversity^1^. Currently, open-access data platforms such as GBIF (Global Biodiversity Information Facility), BOLD (The Barcode of Life), and OBIS (Ocean Biogeographic Information System) provide data spanning genetic, species, and ecosystem scales. These data can be used in a wide range of biodiversity and conservation-oriented analyses aimed at protecting biodiversity in the face of unprecedented human-induced threats^3,4^. However, substantial data biases exist^5^, and most of these platforms do not provide one crucial facet of biodiversity in a standardized way: biotic interactions^6^. A lack of knowledge of species interactions and ecological roles is a major impediment to understanding community function, ecosystem services, and the requirements of species for persistence in a given environment^7–9^.

Biotic interactions interconnect organisms within ecosystems and influence their survival, reproduction, distribution, and abundance^6,10^. Biotic interactions such as competition, predation, parasitism, and mutualism determine species coexistence and shape communities within ecosystems. These interactions contribute to key ecosystem processes such as nutrient cycling and energy flows; therefore, disruptions to these networks can cause negative, cascading impacts on ecosystems^6,10^. Pollinator-plant relationships are a key component of ecosystem function. For conservation-oriented studies, understanding these interactions is essential, because changes in this bidirectional interaction can cause irreversible disruptions to ecosystems^10–12^. Globally, bees (Hymenoptera: Anthophila) are among the most important groups of animal pollinators, playing crucial roles in natural and agricultural ecosystems^13–16^. Recent studies have documented the negative impacts of human activities (habitat loss, agrochemicals, etc.) on these interactions globally^17–19^, though they typically focus on plants or pollinators rather than on their interactions and interdependencies, or they operate at limited scales (but see Biesmeijer *et al*.^20^). Our knowledge of interactions remains largely limited to site-based and local-scale studies in North America and Europe, with relatively few studies in tropical regions^15,21–25^. This pattern likely reflects biases in biodiversity data recording and sharing. Notably, North American studies dominate and largely derive from museum specimens^15^. Accessing and standardizing reliable data on bee-plant interactions is one of the most important and challenging steps in integrating this important ecological facet into biodiversity and conservation studies^1,26^. In light of this, future studies require more findable, accessible, interoperable, and reusable data, as suggested by the FAIR principles^27,28^.

Global Biotic Interactions (GloBI) is an interdisciplinary data indexing service that provides an open-access dataset cataloguing interactions among species (hosts, parasites, vectors, etc.) across the tree of life (www.globalbioticinteractions.org)^6,29^. GloBI documents interactions by integrating and centralizing various ecological interaction data from the scientific literature, previously published datasets, museum collections, and online databases^6,29^. It allows users to query the database interactively for specific organismal interactions^29^ and download the entire database in multiple formats. Most records refer to animals, and the dataset’s biases broadly match that of distributional data, favoring high-income economies^5,29^. Because the dataset draws from a wide range of sources with inconsistent taxonomic nomenclature, substantial data standardization and harmonization are required to make it suitable for downstream research. The resources necessary to conduct thorough normalization for bee occurrence data have only recently become available^30,31^, making bee-focused GloBI possible. The availability of bee interaction data on GloBI is partly a consequence of multiple major National Science Foundation-funded efforts to database bee museum specimens, initiated by the American Museum of Natural History and University of California, Riverside^32^ and continued via the Big-Bee Project and iDigBees^30,33^.

Following FAIR principles, this study streamlines the bee-plant interactions within the GloBI to make them more findable, accessible, interoperable, and reusable for future studies^27,34^. This data paper provides an overview of the quantity and quality of the available data on interactions between bee species and their host plants worldwide. We develop and validate a dataset encompassing 981,982 unique bee-plant interactions involving 5,537 bee species and 12,699 plant taxa, of which 11,163 are identified to species and 1,536 to genus. Furthermore, we highlight potential geographic and taxonomic biases in currently available bee resources (e.g., Discover Life Bee Checklist^35^, Dorey *et al*.^31^) and offer a harmonized, human- and machine-readable dataset for future research. We integrated metadata from multiple resources for continent- and country-level bee distributions and information on human uses of host plants. This validated dataset is a fundamental step toward characterizing interaction data and identifying taxonomic and geographic gaps in our knowledge of bee-plant relationships. We highlight these above-mentioned knowledge gaps to guide future research and data integration efforts, particularly in understudied regions, to support future conservation strategies.

## Methods

### Input data

We began with a raw version of the indexed bee records from the GloBI database (v7.0; https://zenodo.org/records/17957582)^36^, which includes 1,868,618 records representing various bee interactions^6,37^. We first filtered this dataset for bee-plant interactions across the seven bee families (Andrenidae, Apidae, Colletidae, Halictidae, Megachilidae, Melittidae, and Stenotritidae), excluding bee interactions with other organisms. We then formatted the dataset so that bees were always treated as “target taxa” and plants as “host taxa,” removing records with missing values. These steps reduced the original dataset to 986,193 bee-plant interaction records (53% of all the GloBI records involving bees).

Next, we harmonized bee and plant taxonomy through a multi-step process based on the most up-to-date checklists for bees^35,38^ and plants, using the World Checklist of Vascular Plants (WCVP)^39,40^. These checklists provide information about species synonyms, and country- and continent-level distributions for more than 21,000 bee species and all vascular plant species. We included only bees identified to species and plants identified to genus or species. Following taxonomic harmonization, the dataset contained 981,982 unique entries for bees and their host plants. These entries represent interactions of 5,537 bee species with 12,699 plant taxa. Of these plant taxa, 11,163 are identified to species and the remaining 1,536 are identified only to genus. The bee species in the GloBI dataset are connected through 14 interaction types with their host plant, depending mostly on their role in the effective pollination of the visited flower, including: “Interacts with”, “Visits flowers of”, “Pollinates”, etc.

**Fig. 1:**
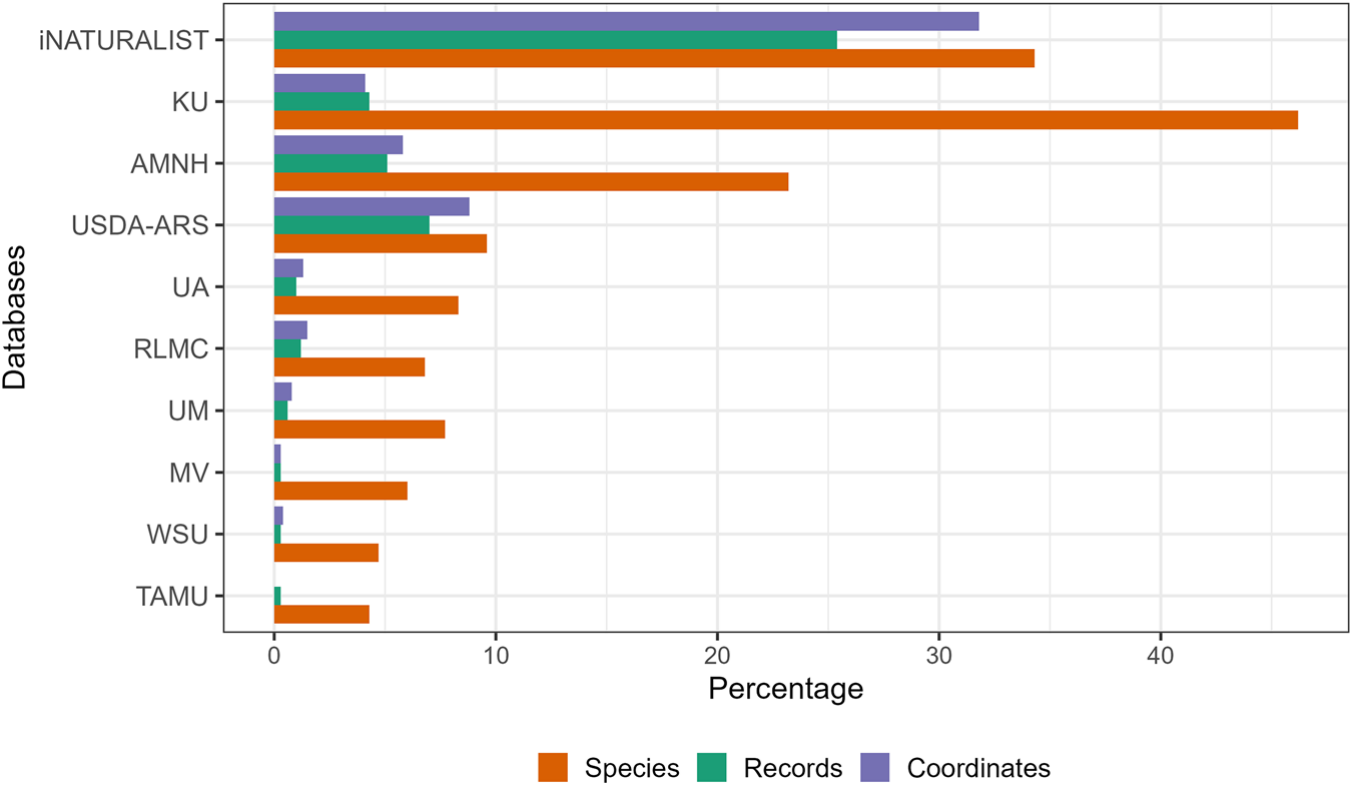
The top 10 institutes with the highest contribution in the GloBI dataset. Bars depict percentage of records (Records), species (Species), and records with coordinates (Coordinates) for bee species in the GloBI dataset across the top 10 institutes: University of Arizona (UA), Insect Collection RL Minckley Insect Collection (RLMC), University of Manitoba J. B. Wallis / R. E. Roughley Museum of Entomology (UM), Bee Specimens from Michael Veit Collection (MV), Frost Entomological Museum, Washington State University (WSU), and Texas A&M University (TAMU).

The GloBI dataset for bee-plant interactions has been compiled from 186 data sources, including research institutions, via data portals and biodiversity repositories, spanning 563,869 items^6,37^ (Fig. 1). The iNaturalist platform alone provides more than 25% of all the records for more than 34% of the species (Fig. 1)^41^. The University of Kansas Natural History Museum Snow Entomological Collection (KU-SEMC) represents more than 46% of the species and 4% of all the records in the dataset^42^ (Fig. 1). Three institutions — KU-SEMC, the American Museum of Natural History (AMNH)^43^, and the U.S. Department of Agriculture Agricultural Research Service (USDA-ARS) Bee Biology and Systematics Laboratory collection (BBSL)^44^ provide more than 16% of all the records for approximately 79% of the species covered. While these datasets provide the majority of data, some sources contain high proportions of regionally unique species not covered by other collections (e.g., the Texas A&M University Insect Collection (TAMUIC)^45^ and Museum of Comparative Zoology, Harvard University (MCZ)^46^. However, more than 32% of records in the dataset lack information about the source institution, many of which likely derive from datasets associated with journal publications.

#### Integrating metadata

**Fig. 2:**
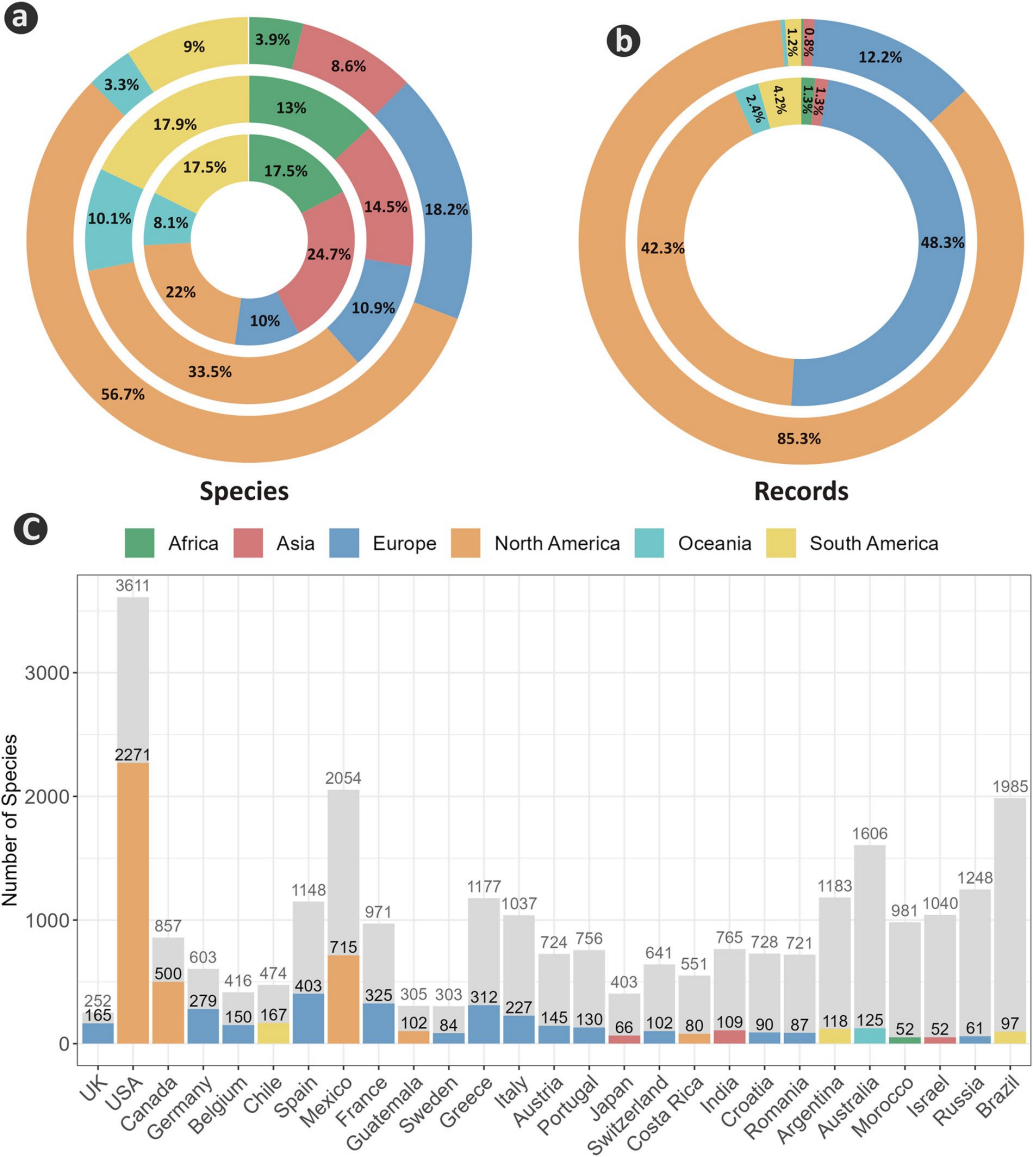
Continental patterns in bee species richness and record volume across three global bee datasets. Donut rings summarize the percentage contribution by continent. The inner ring (**a**) shows bee species richness from the Ascher & Pickering (2024) checklist (>21,000 species). The middle ring (a,b) shows bee species richness (**a**) and occurrence record counts (**b**) from Dorey *et al*. (2023a) (>11,000 species). The outer ring (**a,b**) shows bee species richness (**a**) and georeferenced bee–plant interaction record counts (**b**) from GloBI (4,560 bee species). (**c**) Country-level completeness of bee species richness in GloBI for countries with >50 bee species represented, ordered by coverage rate. Orange indicates species present in GloBI; grey indicates expected species absent from GloBI.

In addition to the GloBI dataset, we used other biodiversity datasets to provide metadata that offer an overview of the global distribution of the bee fauna. We used the updated global checklist of bees from Ascher & Pickering, 2024^35^, embedded in the BeeBDC package (v1.2.1; December 2024)^47^ and its cleaning routines to harmonize the taxonomy of the bee species within the GloBI bee-plant dataset. The Ascher & Pickering, 2024^35^ and Dorey *et al*.^38^ datasets provide the country- and continent-level distribution of more than 21,000 bee species; we used these to compare country and continent coverage of bee species richness (Fig. 2). We also used the most up-to-date version of the dataset by Dorey *et al*.^31^ and 2024^38^, a synthesized global bee occurrence dataset. Through a multi-step data cleaning process, Dorey *et al*.^38^ synthesized a dataset with more than 6.7 million occurrences for more than 11k bee species at the global scale^31^. We used this dataset to compare the species richness and record counts across different continents and countries with the GloBI bee-plant dataset (Fig. 2).

For plants, we used the World Checklist of Vascular Plants (WCVP) package in R (rWCVP, v. 0.5.0)^39,40,48^ to assess the taxonomic validity of host plants in the GloBI bee-plant dataset. Using WCVP and the *taxize* package (v2)^49^, we harmonized host plant taxonomy and replaced synonyms with accepted names^46^. Finally, we extracted data regarding plant species used by humans as food, animal feed, and medicine^50,51^, and integrated that information into our curated dataset. We used these metadata to assess potential biases toward commonly used plant species.

#### A geographical review

We used multiple approaches to highlight the potential geographic biases in the GloBI bee-plant dataset. Of the 981,982 entries in the dataset, around 80% (784,432) include geographic coordinate data. We used longitude and latitude values to assign the continent and country to each record. Following Dorey *et al*.^31^, we flagged coordinate availability as a Boolean field (TRUE/FALSE) to facilitate filtering of records with coordinates. Then, we summarized the number of bee species and records across countries and continents. Next, to investigate geographical bias, we compared species richness and record counts between the global checklist of bees^35,38,47^, and the GloBI bee-plant dataset across countries and continents (Fig. 2).

#### A taxonomic review

**Fig. 3:**
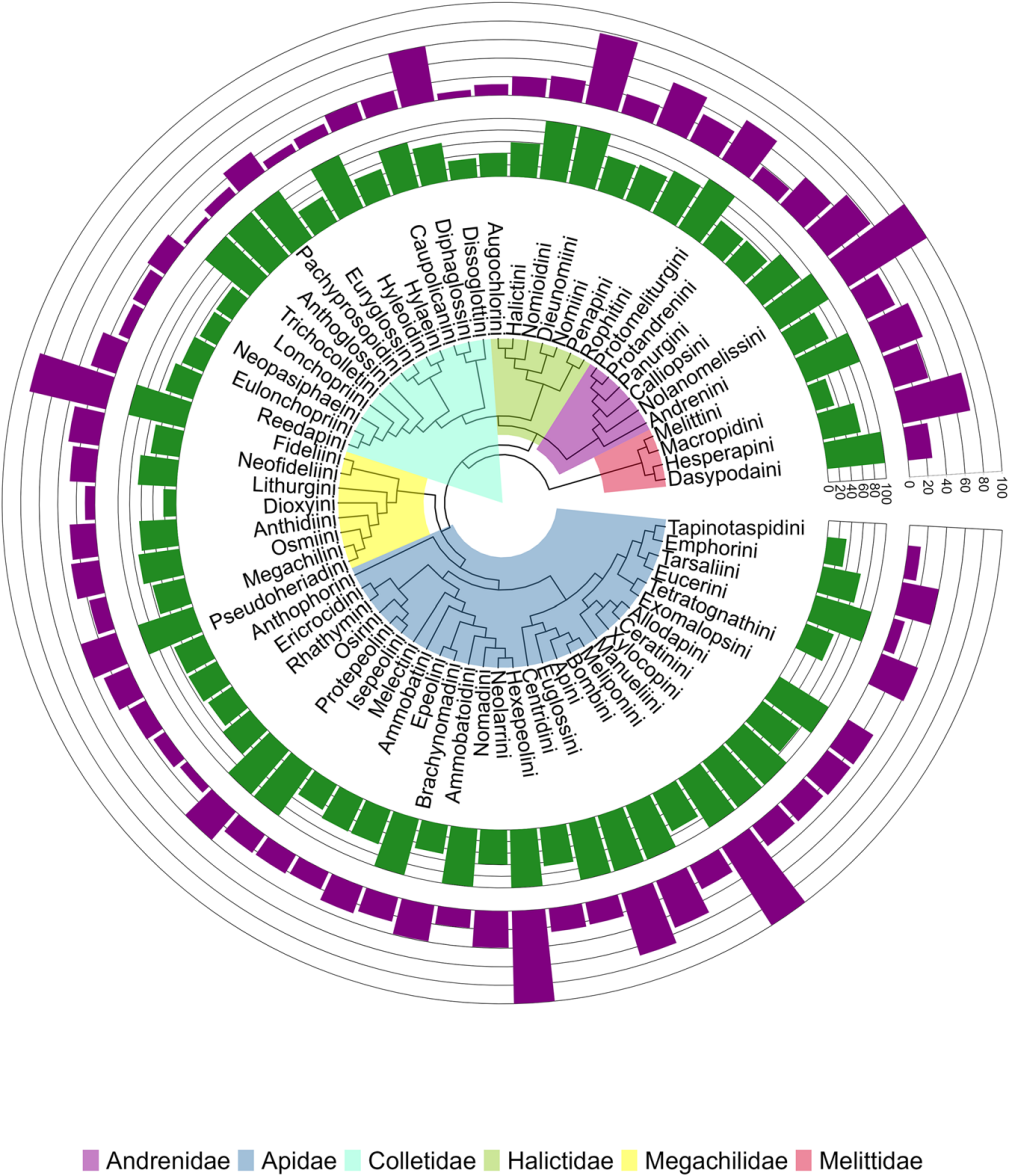
Tribe-level completeness of bee genera and species represented in the GloBI dataset, mapped onto a global bee phylogeny. Purple bars indicate the proportion of species in GloBI relative to the estimated global fauna for each tribe, and green bars indicate the corresponding proportion of genera. The phylogenetic backbone follows the supermatrix phylogeny of the world’s bees (Henríquez-Piskulich *et al*.)^52^.

We explored taxonomic biases in the GloBI bee-plant dataset across multiple taxonomic levels by counting species and records and comparing it with global bee diversity^35,47^. We summarized record counts across different bee species, genera, tribes and families. Using a bee phylogenetic supermatrix^52^, we counted the percentage and number of species and records represented in the dataset for each bee tribe^52^ (Fig. 3).

#### Bee-plant interactions

**Fig. 4:**
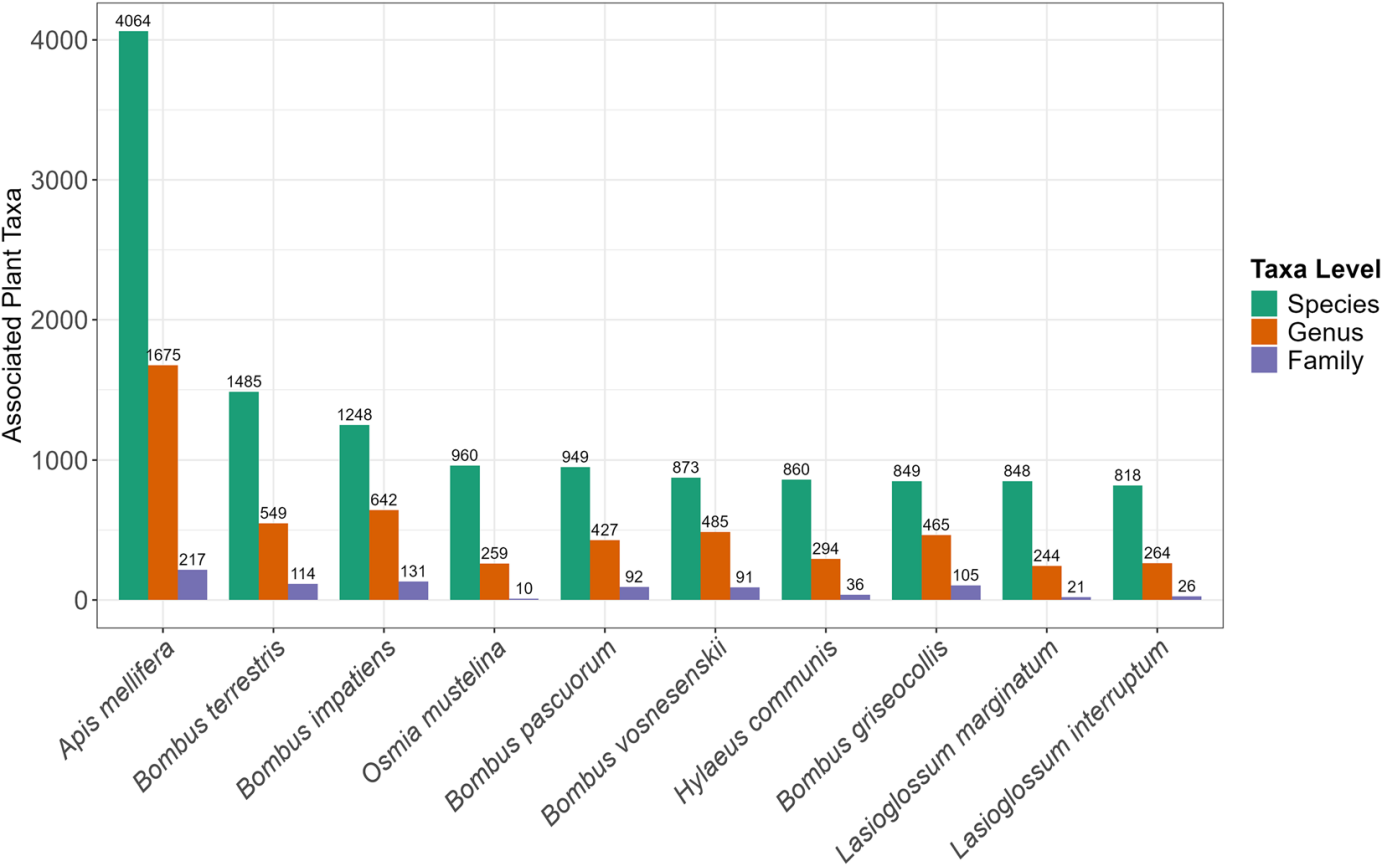
Number of unique plant taxa associated with each of the ten most frequently recorded bee species in the GloBI dataset. For each bee species, bars show the count of unique plant associates summarized at the species (green), genus (orange), and family (purple) levels.

To investigate interactions between bees and plants, we built a pipeline to assess the associations between bee species and plant taxa which, when data are sufficient, can be used to gauge bee species specialization^26^. We also assessed the diversity of associations between bee species and plants by summarizing the number of species, genera, and families of plants per bee species and vice versa (Fig. 4). Given the high proportion of records involving plant species commonly used by humans, we assessed plant-species diversity by use category, including human food, animal feed, and medicine. For this analysis, we included only plants identified to species. Overall, from 11,163 plant species in the GloBI bee-plant dataset, 5,632 (50%) are used by humans for different purposes^47^.

#### Endemism

Finally, we conducted a preliminary assessment of endemism in bee-plant interactions, noting that improved knowledge may reduce the number of apparent endemics. Using the bee checklists^35,47^ and vascular plants (WCVP), we defined endemism at the country level. For plants, we assessed endemism only for records identified to species. We considered a species endemic if it occurred only in one country. We then counted endemic bee and plant species across interactions (Table 1).

**Table 1:** The frequency of endemic bee-plant interactions across the GloBI bee-plant dataset.

Is bee endemic	Is plant endemic	Frequency	Records
Yes	Yes	1,088	3,101
No	Yes	3,353	9,920
Yes	No	7,451	57,093
No	No	127,650	579,549

Where endemism is determined using occurrence records with coordinates and with references to global checklists of bees and plants.

## Data Records

This dataset is a curated subset of bee-plant interaction records from version 7.0 of the Global Biotic Interactions dataset (available at: https://zenodo.org/records/17957582)^36^. We harmonized the taxonomy of both bees and plants using the most up-to-date checklist for each group. For bees, we used two comprehensive datasets, Ascher & Pickering, 2024^35,47^ and Dorey *et al*.^38^, to update taxonomy and add metadata on species distributions. For plants, we used the World Checklist of Vascular Plants (WCVP) was to update the taxonomy of the host plants in the dataset^39,40^. After the multi-step quality control and harmonizing procedures, the dataset contains 981,982 unique records for 5,537 bee species and 12,699 plant taxa. More than 80% of these records include geographic coordinate data. Version 3 of the dataset includes 15 columns describing bee and plant taxonomy, access links, references for each record, and endemism for both bees and plants. We also provide several summaries for bee-plant interactions.

## Data Overview

The GloBI bee-plant dataset covers a modest proportion of total bee diversity (5,537 of 21,436 species; 26%), although North America is well represented, with 2,649 of 5,480 species (48.4%) and 666,628 of 981,982 records (85.3%). With greater shortfalls, only a small portion of all animal-pollinated plants is represented (11,163 out of more than 308k animal-pollinated angiosperms, ~3.6%^53^), mirroring general trends in data availability^15,31^. A comparison with Ascher & Pickering, 2024^34^ shows that the GloBI dataset does not accurately represent many global regions with high bee diversity^15^ (Fig. 2). For instance, although Africa and Eurasia contain more than 55% of described bee species, the GloBI dataset disproportionately represents North America, as noted earlier (Fig. 2). At the continental scale, Asia has more than 6,100 described bee species, but only 8% are included in the GloBI dataset (Fig. 2). Even Europe, which is otherwise relatively well-represented and well-studied for bees, has only approximately 23% of its bee fauna included in the GloBI dataset (851 of 2,486 species; Fig. 2). While North America contains roughly one quarter of global bee species, it accounts for more than 80% of records in bee plant interactions in GloBI and 42% of records in Dorey *et al*.’s dataset.

These trends are also reflected at the country level, where countries across North America, and to a much lesser extent Europe, have a better representation of their fauna in the GloBI bee-plant dataset (Fig. 2a,b). Parts of Europe and North America are relatively well covered relative to other continents. For example, UK includes 65.5% of its bee fauna, the USA includes 63%, Canada 58%, and Germany 46% (Fig. 2c). Conversely, among countries with more than 10 bee species in the GloBI dataset, South Africa, Peru, Türkiye, Ukraine, Kyrgyzstan, Algeria and Pakistan are only represented by less than 3% of their faunas. The USA has the highest number of species in the GloBI dataset. From 3,611 species of bees in the USA, 2,271 (63%) have at least one record in the dataset (Fig. 2c). This pattern holds even when restricting to species with more than 10 unique records in the GloBI dataset, although the numbers are dramatically lower, demonstrating how few species could be considered well documented. The dataset has major geographic biases and highlights gaps in our knowledge of bee distributions, and probably their interactions, particularly across much of Africa and Asia, Central Asia, and South America (Fig. 2). Overall, bee diversity is much higher across the xeric and non-forest areas, concentrated between 20° to 50° latitude in the Northern Hemisphere and −20° to −50° latitude in the Southern Hemisphere.

### Taxonomic bias

Apidae includes 1,843 of 6,207 species (~30%) and has more than 53% of the total records in the GloBI dataset, making it the most represented bee family. It is followed by Andrenidae (1,089 of 3,119 species; ~35%; fourth largest), Megachilidae (1,008 of 4,175 species; ~24%; third largest), Halictidae (916 of 4,504 species; ~20%; second largest), Colletidae (571 of 2,789 species; ~20%; fifth largest), Melittidae (98 of 215 species; ~46%; sixth largest), and Stenotritidae (4 of 21 species; 19%; smallest family; Fig. 3).

The bee tribes Bombini, Apini, and Halictini collectively make up more than 70% of records in the GloBI dataset (Fig. 3), displaying the best coverage in the number of genera and species. The bee genus *Bombus* has the highest number of records in the GloBI dataset (161 of 296 species, ~30% records), followed by *Andrena* (5652 of 1,646 sp., ~8%), *Lasioglossum* (372 of 1,843 sp., ~7%), *Apis* (7 of arguably 11 sp., ~7%), and *Perdita* (275 of 637 sp., ~4%). The remaining genera have less than 4% of all the records in the dataset. Among species, around 7% of all records are from the western honey bee (*Apis mellifera*), followed by the common eastern bumble bee (*Bombus impatiens*; 5%), *Augochlora pura* (2.8%), and *Bombus bifarius* sensu lato (2.2%). Approximately 80% of the records belong to other species of bees in the dataset. Around 29% of the species in the GloBI bee-plant dataset (1,582 species) are single-country endemics; however, they are represented by only 60,538 records (Table 1).

### Interaction between bees and plants

Of the 14 interaction types in GloBI dataset, “interacts with” has the highest number of records (~46%), while there are only four records for “parasite of”. Most interaction types describe bee–flower relationships but use different terminology, likely a simple consequence of different preferences by data publishers. For instance, “pollinates” may very well not always indicate a successful pollination event, but may only indicate the bee visited a flower (see Ollerton *et al*.^54^). Overall, ignoring the interaction types, there are 165,418 unique bee-plant species pairs between bee species and plant taxa in the dataset.

Our dataset is also biased in the number of bee-plant associations. *A. mellifera*, followed by *Bombus terrestris, B. impatiens*, *Osmia mustelina*, *B. pascuorum, and B. vosnesenskii*, have the highest number of associations with host plants at different taxonomic levels. Notably, these are also highly visible species on iNaturalist. Conversely, more than 1,393 bee species are represented by only one record, and more than 2,777 species have fewer than five records. Considering host plants, *Melilotus officinalis, Taraxacum officinale*, and *Rubus sp*. (identified to genus) interact with the highest number of bee species in the GloBI bee-plant dataset, while more than 4,070 plant species interact with only one bee species and 7,500 interact with fewer than five bee species (Fig. 4). Among plants used by humans, the common dandelion (*Taraxacum officinale*) and apple (*Malus domestica*) have the highest number of interactions with bee species. White yarrow (*Achillea millefolium*) and field wild carrot (*Daucus carota*) have the highest number of bee visitors compared with the other plant species used as animal feed.

In our subset of the GloBI dataset, 1,582 of 5,537 (~29%) bee species are nationally endemic (Table 1) and account for 6% of all the records in the GloBI bee-plant dataset. Within our dataset, and at the family level, endemicity was highest in the Andrenidae (344 species), followed by Apidae (283), Colletidae (275), Halictidae (1270), Megachilidae (212), Melittidae (56), and Stenotritidae (2). Conversely, 1,273 plant species in the dataset (11% of plant species in our dataset) are endemic. The frequency of non-endemic bee-plant interactions is the highest (~89%), followed by endemic bees with non-endemic plants (~9%), non-endemic bees with endemic plants (~1.5%), and both endemic (~0.5%).

## Technical Validation

We used the most up-to-date, openly available checklists for bees and plants to harmonize taxonomy^31,35,38–40,47^. These checklists provide the country- and continent-level distributions, which we used to map species distributions and investigate their geographic diversity. We used these resources to define endemism across bee and plant species at national level for further analyses. Additionally, we compared the geographic biases using standardized and curated occurrence dataset by Dorey *et al*.^38^. We also used the most-complete effort phylogenetic tree by Henríquez-Piskulich *et al*.^52^ to investigate the taxonomic biases across the GloBI bee-plant dataset. Finally, we used metadata on anthropogenically relevant plant species to examine bias toward human used taxa^51^. This analysis helped to highlight the frequency of the most common species related to human food production in the dataset. Collectively, these validations show that the dataset is strongly biased; however, it represents a major improvement over existing bee plant data resources and makes biases explicit so users can make informed decisions while also identifying targets for future data integration.

## Usage Notes

This dataset contains substantial improvements necessary to make GloBI ‘fit for use’ in many bee biodiversity analyses at the global scale. Researchers can use this curated bee-plant interaction dataset for a wide range of ecological, biogeographical, and conservation applications. Despite heavy taxonomic and geographic biases, the dataset is valuable and can support further studies. The current version provides a basis for interconnecting these interaction records with synthesized occurrence datasets by Dorey *et al*.^31,38^ to explore the diversity and structure of the bee-plant interaction networks, at least across the well-studied regions such as North America and Europe, and to integrate these data into future conservation-oriented studies. Furthermore, this dataset can be used for defining the patterns of specialization, robustness and modularity in pollination networks globally. The occurrence data can help to ecologically investigate the response of the bee-plant interactions and their co-occurrence under climate gradients and climate change. Results of above-mentioned analyses can be used to define high-diversity regions for bee-plant interactions to delineate higher priority regions for conservation and investigate their environmental and anthropogenic drivers. Finally, integrating functional datasets and pollination methods of the host plants will help to run trait-based research and study bee or plant species regarding their functional roles across different ecosystems.

We recommend that users read this article carefully, consider the limitations we listed, and ensure that additional data are curated using a standard workflow (i.e., the BeeBDC, bdc, and coordinateCleaner pipelines^47,55^) before use to facilitate interpretation. Additionally, users may contact the corresponding authors to report errors or have any suggestions for enhancing the quality of the work. We believe this validated dataset with its metadata will facilitate using the GloBI bee-plant dataset, and the provided pipeline can be used to validate and harmonize future updates.

## Data Availability

The datasets generated and/or analysed during the current study are available at 10.5281/zenodo.18303036 (v. 3.1)^36^.

## Code availability

All code used in this study is available at 10.5281/zenodo.18303036, originally hosted on GitHub. The repository includes scripts for data processing, analysis, and figure generation. Detailed instructions are provided in the README file^36^.
